# A Case of Primary Ciliary Dyskinesia Caused by a Mutation in *OFD1*, Which Was Diagnosed Owing to *Clostridium difficile* Infection

**DOI:** 10.3390/pediatric13020033

**Published:** 2021-05-10

**Authors:** Rina Hasegawa, Shinji Suzuki, Shigeo Nishimata, Yasuyo Kashiwagi, Natsuko Inagaki, Hisashi Kawashima

**Affiliations:** Department of Pediatrics and Adolescent Medicine, Tokyo Medical University, 6-7-1 Nishishinjuku, Shinjuku-ku, Tokyo 160-0023, Japan; shinji-s@tokyo-med.ac.jp (S.S.); s.nishimata@gmail.com (S.N.); hoyohoyo18@hotmail.com (Y.K.); abenatsu@wb3.so-net.ne.jp (N.I.); chop0064@yahoo.co.jp (H.K.)

**Keywords:** ciliary dysfunction, OME, antibiotic exposure, *NODAL*

## Abstract

We report a Japanese 5-year-old boy with primary ciliary dyskinesia (PCD) which was diagnosed owing to *Clostridium difficile* (CD) infection caused by prolonged antibiotic exposure. He had intractable otitis media with effusion (OME) and had abdominal pain and diarrhea for 4 months after starting antibiotics administration. His stool contained CD toxin. After vancomycin treatment, his symptoms improved and his stools did not contain CD toxin. His past medical history included frequent pneumonia. We, therefore, performed electron microscopy of the biopsy specimen from his nasal mucosa and genetic testing, and he was diagnosed with PCD. PCD is a rare inherited genetic disease causing ciliary dysfunction, which is very difficult to diagnose because some children without PCD also develop the same symptoms. Therefore, children who have intractable OME, rhinosinusitis, frequent pneumonia, or bronchitis and are taking antibiotics for long periods of time should be checked for underlying diseases, such as PCD.

## 1. Introduction

Primary ciliary dyskinesia (PCD) is a rare inherited genetic disease causing ciliary dysfunction, which affects 1/20,000 live births [1]. To date, more than 40 genes are known to cause PCD. It is characterized by respiratory infections, sinusitis, and otitis media with effusion (OME). Because children without PCD sometimes also develop these symptoms, PCD is very difficult to diagnose, and a study reported that the majority of participants (70%) had seen a physician more than 50 times before a correct diagnosis was made, taking an average age of 10.91 ± 14.4 years [1]. Herein, we present a case of a patient with PCD, who was diagnosed owing to *Clostridium difficile* infection (CDI) caused by prolonged antibiotic exposure.

## 2. Case Report

**Figure 1:**
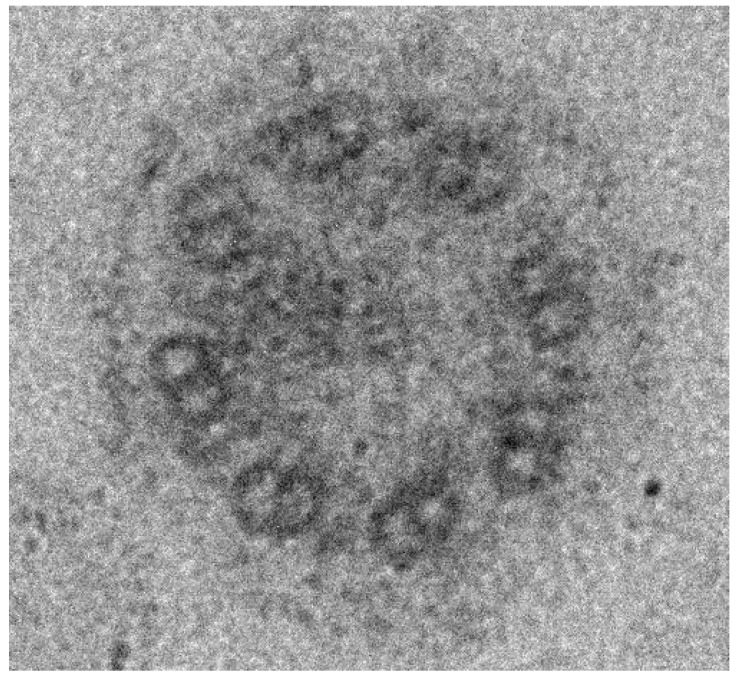
Electron microscopy analysis of the nasal mucosa of the patient. The outer dynein arms are lack-ing in the present patient.

A Japanese 5-year-old boy presented to our hospital with abdominal pain and diarrhea. He had been taking antibiotics since he was 3-years old, because of frequent and intractable OME. His symptoms did not improve, and he underwent an operation for ear-tube insertion in both ears at the age of 4 years. After the operation, his symptoms did not improve, and his otorrhea persisted. A sample of the otorrhea that was taken at the time of the operation and subsequently subjected to microbiological analysis showed the presence of coagulase-negative staphylococci, and he hence started antibiotics treatment again. He was first treated with tosufloxasin, but as his symptoms did not improve, after a few weeks the treatment was changed to clarithromycin, and finally to an amoxicillin-clavulanate combination, but his symptoms still did not improve. Two-months later, he complained of abdominal pain. The abdominal pain became worse, and he had diarrhea about 3 times/day for 4 months after restarting antibiotics administration, and so he was admitted to our hospital for further examination and treatment. He was born at 41 weeks of gestation weighing 3658 g, and was admitted to the neonatal intensive care unit because of meconium aspiration syndrome. Other than the above, his past medical history included frequent pneumonia. Regarding his family medical history, his father had bronchial asthma and his mother had allergic rhinitis. He had no known allergies. He had no fever and his vital parameters were normal. His abdominal pain was more prominent in the left region. He had no vomiting, or bloody stools. Laboratory analysis demonstrated only an increased number of leukocytes and slightly increased erythrocyte sedimentation rate. His intractable OME and frequent pneumonia suggested that he had some type of immune deficiency, such as chronic granulomatous disease or hypogammaglobulinemia. We, hence, analyzed his immunoglobulin levels, lymphocyte subsets by flow cytometry, phagocytic function, and bactericidal activity of neutrophils, but all of these were normal. No cause of infection was found in the bacterial stool cultures, but the stool contained *C. difficile* (CD) toxin. Lower gastrointestinal endoscopy demonstrated no abnormal findings, including pseudomembranous enterocolitis. Pathological analysis of a biopsy specimen from the lower gastrointestinal tract demonstrated no abnormalities. He began oral vancomycin treatment and his symptoms improved after 3 days. His stools did not contain CD toxin after 10 days of vancomycin treatment. From his past medical history of frequent pneumonia and refractory OME, we suspected PCD and performed ciliary biopsy and genetic testing. Electron microscopy of the biopsy specimen from his nasal mucosa showed the absence of dynein arms, which are observed in normal control subjects. (Figure 1) Genetic testing identified NM_003611.2(OFD1): c.2873T > G (p.Leu958Ter) in hemizygote, which has previously been reported to cause PCD [2]. Therefore, the patient was diagnosed with PCD. The exome sequencing demonstrated no other reported variants associated with inborn errors of immunity in this patient. This study was approved by the ethics committee of the Tokyo Medical University and written consent was obtained from his parents.

## 3. Discussion

Cilia are evolutionarily conserved organelles, and motile cilia have a complex (9 + 2) axonemal structure that generates functional ciliary motility. Symptoms of PCD result from variants in genes associated with cilia or ciliary movement. Impaired ciliary motility in embryos results in the *NODAL* gene being randomly expressed in either the right side or the left side of the embryo. When *NODAL* is expressed on the left, the heart develops in the normal location. Expression of *NODAL* on the right results in situs inversus totalis. Decreased ciliary movement results in decreased mucociliary clearance, causing pneumonia, OME, sinusitis, infertility, and ectopic pregnancy [3].

Clinical features of PCD include respiratory distress, pneumonia, rhinorrhea, and nasal obstruction during the neonatal period. In childhood, PCD patients typically have chronic cough with sputum, rhinosinusitis, OME, pneumonia, and bronchiectasis. Women with PCD often have ectopic pregnancies, and half of the men with PCD have fertility problems. In addition, PCD patients may demonstrate heterotaxy syndrome, congenital heart defects, pectus excavatum, and scoliosis. PICADAR is a diagnostic predictive tool for PCD [4]. PICADAR first asks if the patient has a daily wet cough that started in early childhood, because it is specifically designed for patients with chronic respiratory symptoms that started in early childhood. Patients are then asked 7 simple questions in PICADAR, and each answer is given a score between 1 to 4. Patients who were born full term, experienced chest symptoms, such as tachypnea, cough, and pneumonia during the neonatal period, were admitted to the neonatal unit, or have a congenital heart defect, were given 2 points for each item. Patients who have a situs abnormality (situs inversus or heterotaxy) were given 4 points, and those who have perennial rhinitis, or have experienced chronic ear or hearing symptoms, such as glue ear, serous otitis media, hearing loss, and ear perforation, were given 1 point for each item. Patients with a score of greater than 6 are recommended to undergo detailed examination for a diagnosis of PCD. As our patient’s PICADAR score was 7 points, we suspected PCD and performed ciliary biopsy and genetic testing.

According to GeneReviews, the diagnostic criteria for PCD include characteristic findings on electron microscopy or a mutation in a gene known to be associated with PCD, together with typical PCD symptoms [5]. In addition, an international registry for PCD reported that a positive diagnosis is based on typical symptoms with at least 2 abnormal diagnostic tests (high-speed video-microscopy of ciliary motility, immunofluorescence staining of a ciliary biopsy, electron microscopy of a ciliary biopsy, nasal nitric oxide analysis, and genetic testing) [6].

Although fundamental therapies for PCD are presently unavailable, it is important to monitor patients and to provide symptomatic therapies. The PCD Foundation recommends the following [7]: daily chest physiotherapy, exercising to improve mucus clearance, and receiving the recommended vaccinations for the prevention of infections. The most common airway pathogens in children with PCD are *Streptococcus pneumoniae*, *Haemophilus influenzae*, and *Moraxella catarrhalis*, and the PCD Foundation recommends that airway microbiology cultures are performed 2 to 4 times a year. Although antibiotics should be administered for acute respiratory exacerbations in PCD, it is not stated in the recommendations that antibiotics should be administered for sinusitis and OME. However, most PCD patients take antibiotics, and do so for a long time because their symptoms do not improve. Long-term treatment with antibiotics can cause some side effects, such as diarrhea, the appearance of drug-resistant bacteria, and CD infection, as described below.

We identified an *OFD1* gene mutation in our patient. The *OFD1* gene, located on chromosome Xp22.2, has long been recognized as a gene involved in the classic dysmorphology syndrome oral-facial-digital syndrome type I (OFDSI), which causes dysmorphic features of the mouth, face, and digits [8]. Pathogenic variants of *OFD1* were found to be associated with X-linked intellectual disability, Joubert syndrome type 10 (which results in morphological abnormalities of the cerebellum and brainstem), Simpson–Golabi–Behmel syndrome type 2 (which results in macrocephaly, intellectual disability, and obesity), and retinitis pigmentosa. In recent years, multiple phenotypes have been identified as being allelic to OFDSI, including PCD, and some individuals with *OFD1* variants have PCD, either as an apparently isolated phenotype or in combination with other features of *OFD1*-associated disorders. Therefore, we performed brain MRI on this patient.

To date, there have only been a few reports about CD infection in children. Although the high frequency of asymptomatic CD carriers among infants is not well understood, the colonization rate of CD, particularly in newborns and infants younger than 2 years, varies widely among reports (2.5–90%) [9]. Owing to the absence of the cellular pathways necessary for pathogenicity (toxin receptors or downstream signaling pathways), CDI in children is considered to be less frequent and less severe than in adults [10]. However, recently, the incidence of CDI has increased in children who do not have any chronic underlying diseases. Therefore, antibiotics should be used for appropriate periods as required. There are very few reports to date stating that PCD patients develop gastrointestinal complications, including CDI. We believe that our present patient developed CDI owing to long-term antibiotics administration. There are many PCD patients who are undiagnosed, and their intractable OME or rhinosinusitis can result in long-term antibiotics administration. It is hence possible that PCD is diagnosed in patients owing to CDI. Therefore, children who are taking antibiotics for long periods of time for intractable OME, rhinosinusitis, frequent pneumonia, or bronchitis should be checked for underlying diseases, such as PCD.

## Data Availability

Not applicable.
